# The positive effect on ketamine as a priming adjuvant in antidepressant treatment

**DOI:** 10.1038/tp.2015.66

**Published:** 2015-05-26

**Authors:** A Melo, N Kokras, C Dalla, C Ferreira, A P Ventura-Silva, N Sousa, J M Pêgo

**Affiliations:** 1 Life and Health Sciences Research Institute (ICVS), School of Health Sciences, University of Minho, Braga, Portugal; 2 ICVS/3B's - PT Government Associate Laboratory, Braga/Guimarães, Portugal; 3 Department of Pharmacology, Medical School, University of Athens, Athens, Greece

## Abstract

Ketamine is an anesthetic with antidepressant properties. The rapid and lasting effect of ketamine observed in preclinical and clinical research makes it a promising therapeutic to improve current major depression (MD) treatment. Our work intended to evaluate whether the combined use of classic antidepressants (imipramine or fluoxetine) and ketamine would improve the antidepressant response. Using an animal model of depressive-like behavior, we show that the addition of ketamine to antidepressants anticipates the behavioral response and accelerates the neuroplastic events when compared with the use of antidepressants alone. In conclusion, our results suggest the need for a reappraisal of the current pharmacological treatment of MD.

## Introduction

Major depression (MD) is a highly incident and prevalent multifactorial disease responsible for great personal and socioeconomic burden.^1^ Currently used therapeutic regimens have important limitations such as long-time lag for clinical response, induction of resistance or even response failure.^2, 3^ Several different factors seem to have a role in MD: imbalances in neurotransmitters^4^ and cytokine production,^5, 6^ hypothalamic–pituitary–adrenal axis dysregulation^7^ and impaired neuroplasticity.^8, 9^

Currently used antidepressants target the metabolism/transport of monoaminergic neurotransmitters, such as serotonine and noradrenaline. However studies show that glutamate (GLU) deregulation is also implicated in MD.^10, 11^ The use of *N*-methyl-D-aspartate (NMDA) receptor antagonists as antidepressants is a relatively new concept.^12^ Ketamine (KET), an NMDA antagonist, was observed to induce a rapid antidepressant action in MD patients,^13^ even in those considered treatment resistant.^14, 15^ The rapid antidepressant effect of KET has also been described in animal models of depressive-like behavior.^16, 17^ Such an effective drug promises to be an important therapeutic tool to improve MD treatment efficacy but, surprisingly, studies where classic antidepressants are used in combination with NMDA antagonists are scarce. Therefore, herein we assessed whether the use of an acute KET treatment could improve the efficacy and/or anticipate the clinical effect of both tricyclic antidepressants and selective serotonin reuptake inhibitor agents in a rat model of depression.

## Materials and methods

### Animals

**Figure 1:**
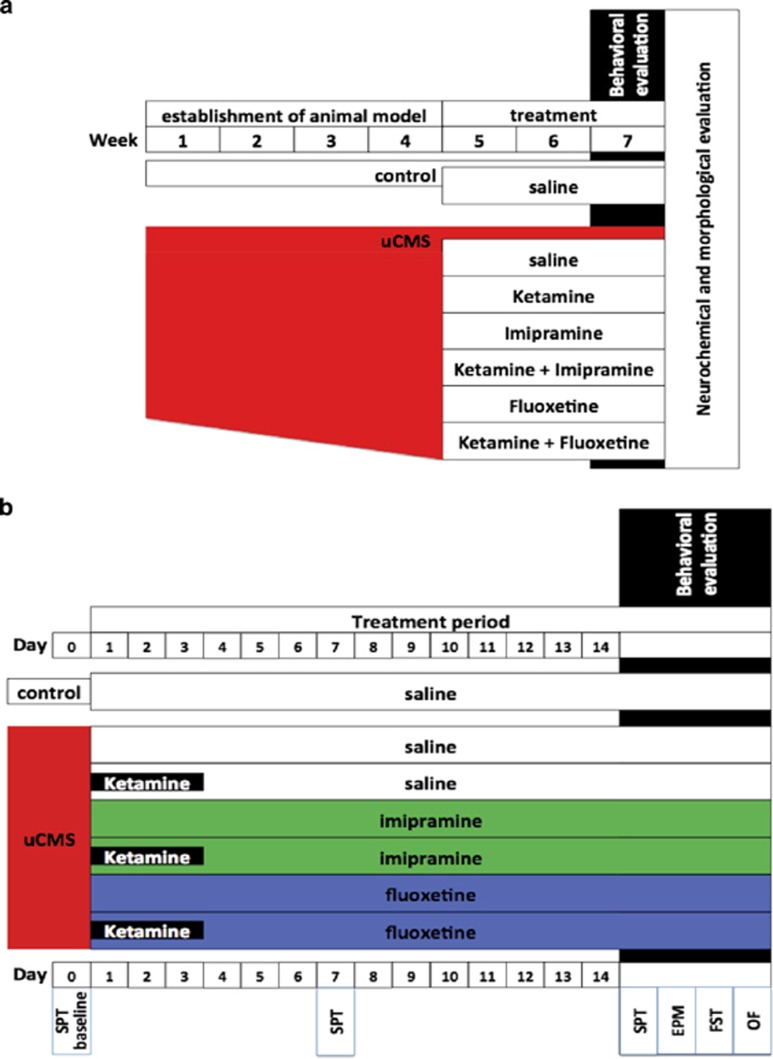
Diagram with experiment protocol. Complete experiment protocol (**a**); treatment period and behavioral evaluation (**b**). EPM, elevated-plus maze; FST, forced-swim test; OF, open field; SPT, sucrose preference test; uCMS, unpredictable chronic mild stress.

Male Wistar rats (Charles River Laboratories, Barcelona, Spain), aged 3 months were housed (three per cage) under standard laboratory conditions (12 h light: 12 h dark cycle, at 22 °C, relative humidity of 55% free access to food and water). Eighty-four animals were used. Animals initially performed the determination of the baseline value of sucrose consumption test (SCT) and then were randomly assigned to the following experimental groups—a control group without stress exposure and six groups exposed to unpredictable chronic mild stress (uCMS). After 4 weeks used to establish a depressive-like behavior phenotype, uCMS-exposed animals were randomly assigned to six different groups: one untreated group (i) uCMS, and the other five experimental groups according to the assigned treatment: (ii) imipramine, (iii) fluoxetine, (iv) KET alone for the first 3 days and saline for the remainder period (uCMS-KET), or treated with KET daily for 3 days and either (v) FLX (KET-FLX) or (vi) IMP (KET-IMP) for the remaining treatment period (*n*=12 per group; Figure 1).

Behavioral tests were conducted during the diurnal phase, between 1000 and 1800 h. Animals were euthanized 24 h after performing the last behavioral evaluation and brains processed for neurochemical and structural analysis. All the procedures were carried out in accordance with the Animal Ethics Committee of the Portuguese National Veterinary Directorate and with the guidelines for the care and handling of laboratory animals in the Directive 2010/63/EU of the European Parliament.

### Unpredictable chronic mild stress

A modified version of an uCMS protocol^18^ was used to establish the animal model of depressive-like behavior. It consisted of chronic exposure to unpredictable mild stressors (confinement to a restricted space for 1 h, placement in a tilted cage (30 °) for 3 h, housing on damp bedding for 8 h, overnight illumination, 18 h food deprivation followed by exposure to inaccessible food for 1 h, water deprivation for 18 h followed by exposure to an empty bottle for 1 h, and reversed light/dark cycle for 48 h every 7 days) until establishment of the model phenotype and then through the duration of behavioral evaluation.

### Antidepressant treatment

After establishment of depressive-like behavior, uCMS animals were treated according to the assigned group with daily intraperitoneal injections of saline, FLX (10 mg kg^−1^, Kemprotec, Cumbria, UK), IMP (10 mg kg^−1^, Sigma-Aldrich, St. Louis, MO, USA) and KET (10 mg kg^−1^, Pfizer, New York, NY, USA) with 0.9% saline as vehicle for 14 days and then performed behavioral evaluation. Treatment and administration scheme were chosen on the basis of their therapeutic effects in previous studies.^8, 19^

KET is a general anesthetic with psychomimetic characteristics and has been used to induce psychotic trait in animal models of schizophrenia. To rule out that the dosage-induced phenotypic changes related no animal models of schizophrenia, we assessed locomotor activity in the open field and sensorimotor gating in the pre-pulse inhibition (PPI).

### Behavioral analysis

All animals (controls and uCMS-exposed) were submitted to a series of behavioral testing. Sucrose preference tests were performed weekly over the 6 weeks of exposure to uCMS. The remainder tests (elevated-plus maze, forced-swim test and open field) were performed after 14 days of treatment (Figure 1).

### Sucrose consumption test

Anhedonia was assessed weekly during exposure to uCMS using the SCT. Briefly, animals were allowed to habituate to the sucrose solution for 1 week before the uCMS protocol to establish baseline preference levels. To test sucrose preference, animals that were food- and water-deprived for 18 h were presented with two pre-weighed bottles containing 1% sucrose solution or tap water for a period of 1 h. Sucrose preference was calculated according to the formula: sucrose preference=(sucrose intake/(sucrose intake+water intake)) × 100, as previously described.^8^ Anhedonia was defined as a reduction in sucrose preference.

### Elevated-plus maze

To assess anxiety-like behavior, animals were tested on an elevated-plus maze (MED-NIRPMNR; Med Associates, St Albans, VT, USA) as previously described.^20^ Animals were placed in the central junction facing an open arm, and allowed to explore for 5 min. Entry was defined as all four paws being positioned within one arm. The test was recorded and the ratio between time in open arms and time in closed arms was measured. Activity in the open arms was calculated as open arm entries percentage (entries into the open arms/total entries into all arms) and time spent in open arm percentage (time spent in the open arms/total time spent in all arms). The degree of anxiety was indirectly related to the time spent in the open arms and the number of open arm entries.

### Forced-swim test

Learned helplessness was evaluated in the forced-swim test on the last day of exposure to uCMS. Twenty-four hours after a pre-test session (10 min), rats were placed in cylinders filled with water (25 °C; depth 30 cm) for a period of 5 min. Test sessions were assessed using a camera connected to a video tracking system (Viewpoint, Lyon, France); the system automatically calculated immobility time and latency to immobility. Learned helplessness behavior was defined as an increase in time of immobility and a decrease in latency to immobility.

### Open field

To assess locomotor activity, rats were placed in an open-field apparatus (43.2 (length) × 43.2 (width) × 30.5 (height) cm, transparent acrylic walls and white floor, Med Associates) in a room illuminated by white light. Instant position was monitored over a period of 5 min by an array of two 16 beam infrared arrays. Total distance and average speed was used as a measure of locomotor activity.

### Pre-pulse inhibition

An additional setting of animals was used to evaluate KET safety regarding induction of PPI impairment. Startle reflexes were measured in two identical startle response systems (SR-LAB, San Diego Instruments, San Diego, CA, USA), each consisting of a non-restrictive Plexiglas cylinder (8.8 (internal diameter) cm, 22.2 (length) cm), mounted on a Plexiglas platform and placed in a ventilated, sound-attenuated chamber. Cylinder movements were detected and measured by a piezoelectric element mounted under each cylinder. A dynamic calibration system (San Diego Instruments) was used to ensure comparable startle magnitudes across the two devices. Startle stimuli were presented through a high frequency speaker located 33 cm above the startle chambers. Startle magnitudes were sampled each millisecond (ms) during a period of 200 ms beginning at the onset of the startle stimulus. A startle response is defined as the peak response during this 200-ms period. Animals were habituated to the apparatus 5 min daily 2 days before the testing period. After the habituation period, rats were taken from their home cage and placed in the test chamber. The chamber was then sealed and each animal allowed to acclimatize to the startle chamber for a period of 5 min. In addition, background white noise, with an intensity of 63 dB, was maintained to minimize the impact of acoustic stimuli outside the chamber environment. Following five introductory 120-dB startle trials (noise lasting 40 ms), a total of 35 test trials were pseudo-randomly delivered as follows: (a) five trials with background noise only, (b) 10 startle trials of 120 dB and (c) five pre-pulses of each of four different intensities preceding a startle trial. Pre-pulse intensities of 2, 4, 8 and 16 dB above the background noise level lasted 20 ms and preceded the 120-dB startle presentation in 100 ms. Inter-trial intervals ranged from 10 to 20 s. The average startle response was assessed in the 100-ms period following the onset of the startle stimulus presentation.

### Neurochemical analysis

Monoamines levels were measured using high performance liquid chromatography (HPLC), combined with electrochemical detection. From each experimental group, seven rats were killed by decapitation. After decapitation, the animal heads were snap-frozen in liquid nitrogen to prevent tissue degradation. The brains were quickly extracted and placed in an icy surface; under a stereomicroscope the prefrontal cortex (PFC), dorsal hippocampus, ventral hippocampus and nucleus accumbens (NAc) were dissected. The dissected tissues were weighed and then homogenized and deproteinized in 100 μl of 0.2 N perchloric acid solution (Applichem, Darmstadt, Germany) containing 7.9 mM Na_2_S_2_O_5_ and 91.3 mM Na_2_-EDTA (Riedel-de Haën, Seelze, Germany), centrifuged at 15 000 r.p.m. for 45 min at 4 °C and the supernatant stored at −80 °C until analysis.

The analytical measurements were performed using a GBC LC1150 (GBC, Braeside, VIC, Australia) HPLC pump coupled with a BAS LC4C (Bioanalytical Systems, West Lafayette, IN, USA) electrochemical detector and pre-column derivatization as described previously.^21^ The working electrode was glassy carbon, the reference electrode was Ag/AgCl and the columns used were ODS Hypersil, 250 mm × 4.6 mm, 5 μm (Thermo Fisher Scientific, Waltham, MA, USA). The voltage of the working electrode was set at +800 mV in the LC4C amperometric detector and the flow rate of the LC1150 HPLC pump was set at 1.0 ml min^−1^. The mobile phase consisted of an acetonitrile (Merck, New York, NY, USA): 100 mM phosphate buffer (5:95) pH 4.9, containing 50 μM Na_2_-EDTA (Riedel-de Haën). Samples were initially diluted 1:5 with ddH_2_O, then further diluted 1:1 with 0.1 M Borax buffer (Sigma-Aldrich), pH 9.6. o-Phthalaldehyde (Sigma-Aldrich) was subsequently added and left to react at room temperature for 10 min before injection. Quantification of GLU and aspartate (ASP) was done by comparison of the area under the curve with that of reference external standards using HPLC software (Clarity, Data-Apex, Prague, Czech Republic), as previously described.^22, 23^

### Histological procedures

After the end of behavioral evaluation, five rats from each group were perfused transcardially with saline (NaCl 0.9%) under deep pentobarbital anesthesia. Brains were removed and kept in Golgi-Cox solution for 15 days and then transferred to a 30% sucrose solution for 5 days. Sections (200 μm) were obtained using a vibratome and collected in 6% sucrose and blotted dry onto gelatin-coated microscope slides. They were alkalinized in 18.7% ammonia, developed in Dektol (Kodak, Rochester, NY, USA), fixed in Kodak Rapid Fix, dehydrated and xylene-cleared before coverslipping. Dendritic arborization and spine numbers were analyzed in layer II/III of infralimbic (IL) area of PFC, and dentate gyrus and CA3 region of hippocampus. Selected neurons had every branch of the dendritic tree reconstructed at × 1000 (oil) magnification using a motorized microscope (Axioplan 2; Carl Zeiss, Jena, Germany) and Neurolucida software (Microbrightfield, Williston, VT, USA) and three-dimensional analysis of the reconstructed neurons was performed using NeuroExplorer software (Microbrightfield). For each animal, 20 neurons were studied and measurements from individual neurons from each animal were averaged. The following dendritic morphology parameters were examined: dendritic length and the number of primary dendrites and dendritic branching points were compared across experimental groups; dendritic spines were assessed according to their morphology mushroom-shaped, thin, thick and ramified spines and dendritic spine density (number of spines/dendritic length) and the proportion of spines in each category was calculated for each neuron in branches that were either parallel or at acute angles to the coronal surface of the section. In dentate granule cells, proximal and distal branches were analyzed for each neuron; basal branches and proximal and distal apical branches in pyramidal neurons in the CA3 region and IL area of the PFC were analyzed. Neuronal reconstructions were masked to the observer.

### Statistical analysis

Sample size was determined considering a medium effect size (*f*=0.25), a type I error *α*=0.05 and a statistical power (1—type II error) of 0.8, and also the performance of HPLC and Golgi analysis after behavioral analysis, with the need to establish two diferent groups of animals. In practice, we ended up using a total of 84 animals, with *n*=12 to each experimental group.

During the experiment, we performed three moments of randomization: the first one after the establishment of a baseline in SCT, the second one after establishment of the uCMS phenotype, and the last one after behavioral evaluation to establish two separate groups to perform either HPLC or neurostructural analysis.

We used masking of the experimental group assignment to all animal/samples during performance and analysis of behavior tests, HPLC and structural Golgi analysis.

Data obtained in experiment were analyzed applying analysis of variance. Quantitative data obtained for parameters analyzed regarding elevated-plus maze, forced-swim test, open field, PPI, neuronal dendritic architecture and HPLC measurements were analyzed using one-way analysis of variance, and whenever appropriate, *post hoc* comparisons between experimental groups were performed using Bonferroni's test. For data obtained in the SCT, we used repeated measures analysis of variance. Sphericity assumption and homogeneity of group variances were verified and statistical analyses were made accordingly. During statistical analysis, we did not perform any data transformation. All data are presented as means±s.e.m. In all cases, statistical significance was set at *P*⩽0.05 (two-sided). Statistical analysis was performed using IBM SPSS statistics 20.0 (IBM, Armonk, NY, USA).

## Results

### Behavioral data

**Figure 2:**
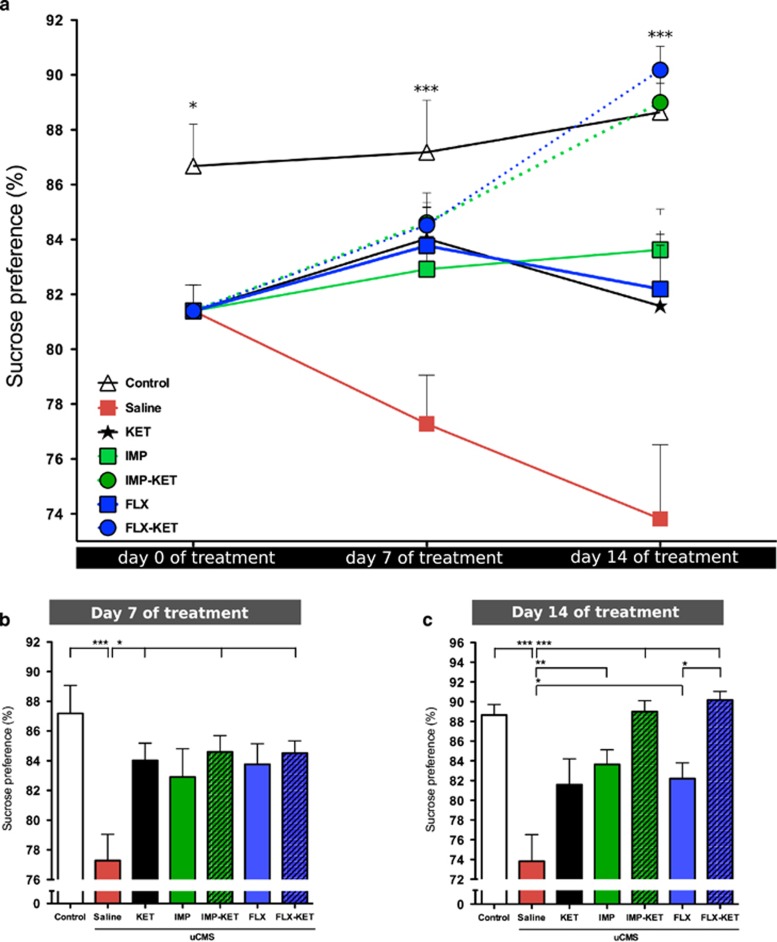
Ketamine effect in the reversion of anhedonic phenotype. Effect of uCMS in sucrose preference (**a**). Reversion of anhedonic phenotype at the end of first week of treatment in KET, FLX-KET and IMP-KET (*P*<0.05) (**b**); rescue of anhedonic behavior by the end of second week of treatment in all animals treated with antidepressant and the addition of KET produces a significantly higher sucrose preference by the end of the second week when comparing the group FLX-KET with FLX (*P*<0.05) (**c**). FLX, fluoxetine; IMP, imipramine; KET, ketamine; uCMS, unpredictable chronic mild stress. Mean±s.e.m., *n*=12, *P<0.05; **P<0.01; ***P<0.001.

To evaluate hedonic behavior, the SCT was performed weekly during the establishment of the depressive-like behavior and during the treatment period. After 4 weeks of exposure to uCMS, animals developed a significant difference in sucrose preference compared with control animals (*P*<0.05). On the seventh day of treatment, animals treated with KET (*P*<0.05), FLX-KET (*P*<0.05) and IMP-KET (*P*<0.05) animals (F_6,77_=4.179, *P*<0.01), but not the remaining treated groups had reversion of anhedonic phenotype (Figure 2b). On the 14th day, all animals treated with antidepressant display behavioral rescuing of anhedonic behavior (IMP (*P*<0.01) and FLX (*P*<0.05), IMP-KET (*P*<0.001) and FLX-KET (*P*<0.001)), except for those given only KET that did not display any longer the behavioral rescuing effect detected 1 week before (F_6,77_=10.50, *P*<0.001; Figure 2c). In animals given FLX, when KET was added (FLX-KET) there was a significantly higher sucrose preference at 14th day when compared with animals given FLX alone (Figures 2a and c).

**Figure 3:**
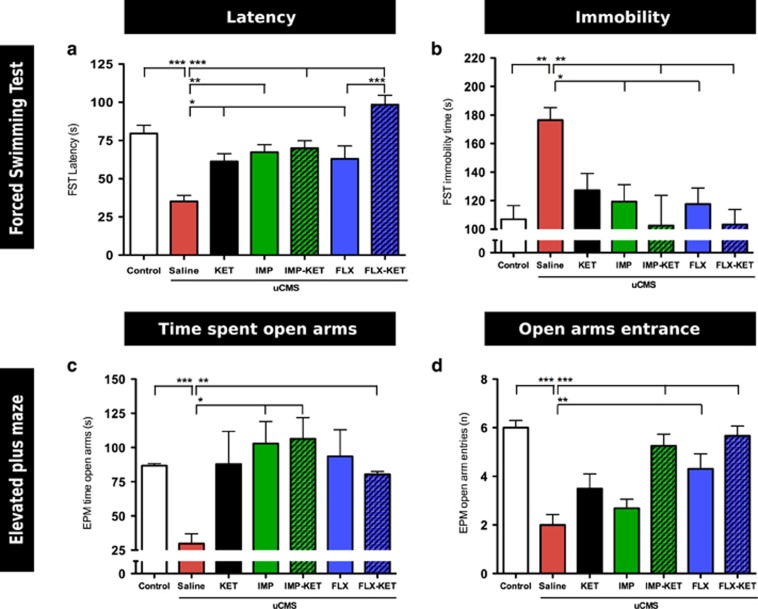
Ketamine treatment effect in anxiety and learned helplessness. All treated animals showed a significantly increased latency to immobility: FLX, KET (*P*<0.05), IMP, (*P*<0.01) and IMP-KET, FLX-KET (*P*<0.001). The addition of KET caused a significant increase in latency to immobility time in FLX-KET against KET (**a**); significant decrease in immobility time: IMP, FLX (*P*<0.05) and IMP-KET, FLX-KET (*P*<0.01) (**b**); IMP-KET (*P*<0.05) and FLX-KET (*P*<0.01) groups with significant increase in time spent in open arms when compared with uCMS animals (**c**); FLX (*P*<0.005), IMP-KET (*P*<0.001) and FLX-KET (*P*<0.001) with significant higher entries in open arms (**d**). FLX, fluoxetine; IMP, imipramine; KET, ketamine; uCMS, unpredictable chronic mild stress. Mean±s.e.m., *n*=12, *P<0.05; **P<0.01; ***P<0.001.

In the forced-swim test, uCMS animals showed decreased latency to immobility time (F_6,77_=7.912, *P*<0.001) and increased immobility time (F_6,77_=11.00, *P*<0.01) when compared with control animals. Comparing with uCMS, all treated animals showed a significantly increased latency to immobility: FLX, KET (*P*<0.05), IMP (*P*<0.01) and IMP-KET, FLX-KET (*P*<0.001; Figure 3a); and also a significant decrease in immobility time: IMP, FLX (*P*<0.05) and IMP-KET, FLX-KET (*P*<0.01; Figure 3b). In latency to immobility time, the addition of KET to those animals treated as well with FLX induced a significant beneficial effect.

In the elevated-plus maze test, uCMS animals displayed an anxious phenotype, spending significantly less time exploring open arms than control animals (F_6,77_=3.111, *P*<0.001); both IMP-KET (*P*<0.05) and FLX-KET (*P*<0.01) groups showed a significant increase in time spent in open arms when compared with uCMS animals; no other significant differences were found (Figure 3c). Regarding the number of entries in open arms, control animals showed a significantly higher number than untreated uCMS group (F_6,77_=11.05, *P*<0.001) and both FLX (*P*<0.01) and IMP-KET, FLX-KET (both *P*<0.001) also have significantly higher entries in open arms (Figure 3d).

No significant effects of uCMS or pharmacologic treatment were found concerning locomotory activity, as evaluated in the open-field test (data not shown). Treatment with KET, in the dosage used in our experimental protocol, failed to show PPI disruption (data not shown).

### Neurotransmitter profile

**Figure 4:**
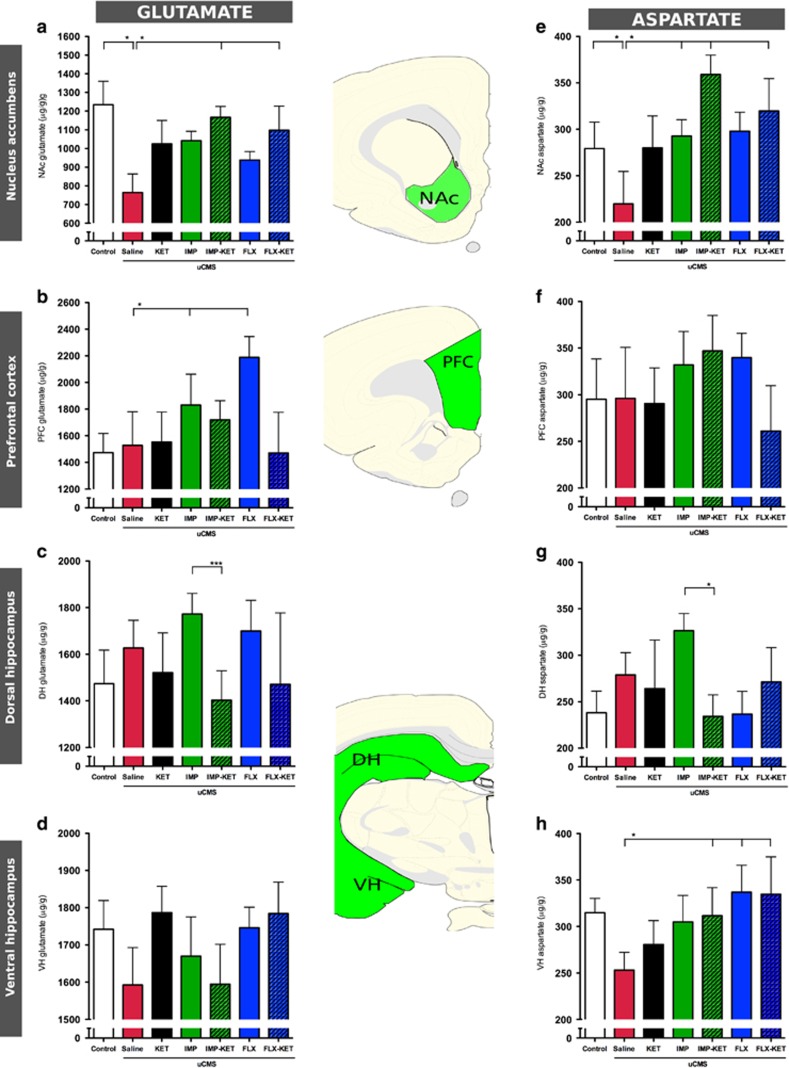
Ketamine treatment induces changes in HPLC measured levels of (**a**–**d**) glutamate (GLU) and (**e**–**h**) aspartate. IMP-KET and FLX-KET increase in GLU levels compared with untreated animals in NAc (both *P*<0.01) (**a**). Treatment in IMP-KET and FLX-KET increases aspartate levels in PFC (*P*< 0.05) (**f**). FLX, fluoxetine; IMP, imipramine; KET, ketamine; NAc, nucleus accumbens; PFC, prefrontal cortex. Mean±s.e.m., *n*=7, *P<0.05; ***P<0.001.

In MD, therapy is still largely based on the ‘monoaminergic hypothesis', which is fundamentally associated with alterations in the level of neurotransmitters. According to this and as an attempt to investigate the possible mechanism underlying the effect of giving KET together with antidepressants, the levels of neurotransmitters, more specifically GLU and ASP were analyzed by HPLC. For that, important key areas for MD such as PFC and hippocampus were selected. In addition, we have investigated the levels of these neurotransmitters in additional brain areas, which are related to the behavioral profile of recovery, such as the NAc. (Figure 4).

Taking into consideration the GLU levels, NAc was the only stress-induced brain region that displayed a reduction in its levels (F_6,55_=2.254, *P*<0.01). Treatment with antidepressants produced an increase in GLU levels, but only IMP-KET and FLX-KET displayed a significant increase in GLU levels compared with untreated animals (both *P*<0.01; Figure 4a).

Measurement of ASP levels showed no overall effect in PFC (F_6,55_=0.672, *P*=0.673; Figure 4f), dorsal hippocampus (F_6,55_=1.397, *P*=0.234; Figure 4g) and ventral hippocampus (F_6,55_=1.143, *P*=0.325; Figure 4h). Contrarily, in the NAc brain area, there was an overall group effect (F_6,55_=2.554, *P*=0.031); *post hoc* analysis showed control animals have higher ASP than uCMS untreated animals (F_6,55_=2.261, *P*<0.05) and that uCMS-exposed animals treated with IMP-KET and FLX-KET have significantly higher ASP levels than untreated uCMS animals (F_6,55_=2.261, *P*< 0.05; Figure 4f).

### Neuronal structural analysis

Given the neuroplastic action of antidepressants, we thought it to be interesting to analyze dendritic architecture of neurons in these areas. There is evidence that KET can induce rapid changes in dendritic spine morphology, which may be relevant to the antidepressant effect shown in the behavioral analysis. We focus our attention in neurons from layers II/III of IL cortex in PFC, pyramidal neurons from CA3 and granule neurons from the hippocampus and medium spiny neurons from NAc.

**Figure 5:**
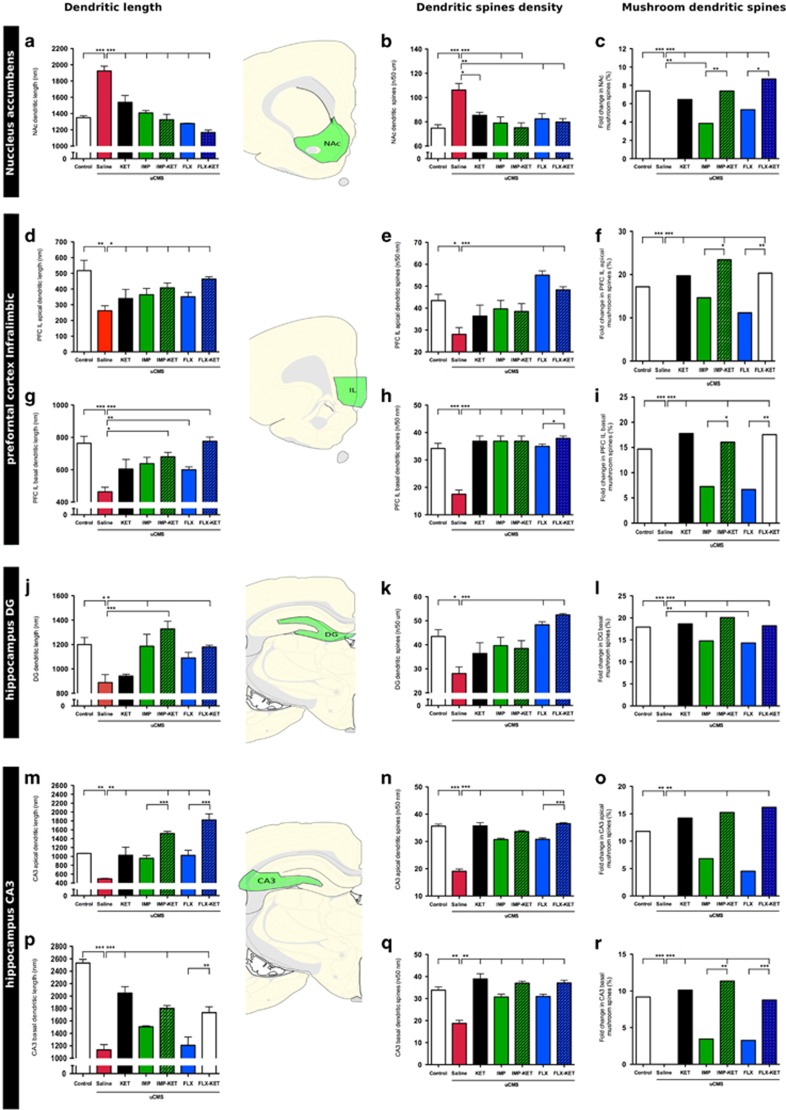
Addiction of ketamine induces changes in neuronal dendritic morphology. In NAc medium spiny neurons: all treatment groups restored decreased dendritic length (all *P*<0.01) (**a**); increase in SD in IMP, IMP-KET, FLX-KET (*P*<0.01) and FLX (*P*<0.05) treatment groups (**b**); increase of MS population (IMP-KET and FLX-KET) compared with the animals given only antidepressant (IMP and KET) (**c**). IL apical dendrites recover from decreased dendritic length (**d**). In IL apical dendrites, FLX (*P*<0.001) and FLX-KET (*P*<0.001) increased (**e**). IL basal dendrites, treatment with IMP-KET (*P*<0.01), IMP (*P*<0,05) and FLX-KET (*P*<0,001) induced a reversion of dendritic shortening (**g**). In IL basal dendrites, FLX-KET increased SD over FLX alone (**h**). Both IMP-KET and FLX-KET have increase in MS population in apical and basal dendrites when compared with IMP (*P*<0.05) and FLX (*P*<0.01) alone (**f** and **i**). In DG hippocampal neurons: IMP-KET (*P*<0.001), IMP and FLX-KET (both *P*<0.05) reverse the shortening in DG dendritic length (**j**); FLX and FLX-KET (*P*<0.001) increased SD (**k**); KET (*P*<0.001), IMP (*P*<0.01), IMP-KET (*P*<0.001), FLX (*P*<0.01) and FLX-KET (*P*<0.001) restored MS (**l**). In hippocampal CA3 pyramidal neurons: KET (*P*<0.001), IMP-KET (*P*<0.001) and FLX-KET (*P*<0.001) recovered basal dendritic length and FLX-KET produced increase in basal dendritic length compared with FLX alone (*P*<0.01) (**p**); KET (*P*<0.05), IMP-KET (*P*<0.001), FLX (*P*<0.05) and FLX-KET (*P*<0.001) induced recovery in apical dendrites, both IMP-KET and FLX-KET promoted a higher regrowth than IMP (*P*>0.001) and FLX (*P*>0.001) (**m**). Addition of KET to FLX treatment increases SD (*P*<0.001) in apical dendritic tree (**n**) and KET (*P*<0.01), IMP-KET (*P*<0.01) and FLX-KET (*P*<0.01) increased the amount of MS (**o**); In CA3 basal dendrites all treated groups had a increase in SD (**q**); treatment with KET (*P*<0.001), IMP-KET (*P*<0.001) and FLX-KET (*P*<0.001) produced an increase in MS and IMP-KET (*P*<0.01) and FLX-KET (*P*<0.001) induce higher MS population than IMP and FLX (**r**). DG, dentate gyrus; FLX, fluoxetine; IMP, imipramine; KET, ketamine; MS, mushroom spine; NAc, nucleus accumbens; SD, spinal density. Mean±s.e.m., *n*=5, *P<0.05; **P<0.01; ***P<0.001.

In the pyramidal neurons of the IL, uCMS induced atrophy in basal and apical dendrites (F_6,28_=8.563, *P*<0.001; F_6,28_=4.074, *P*<0.01). In basal dendrites, treatment with IMP-KET (*P*<0.01), FLX (*P*<0.05) and FLX-KET (*P*<0.001) induced a reversion of dendritic shortening. In the apical dendrites, all treated animals (*P*<0.05) had a significant recovery (Figures 5g and d).

In dentate gyrus granule cell dendrites, data confirm a significant stress-induced atrophy (F_6,28_=7.103, *P*<0.05), which was recovered by the treatment with IMP-KET (*P*<0.001), IMP and FLX-KET (both *P*<0.05). Animals treated with KET alone and FLX alone did not show significant changes in dendritic length (Figure 5j).

Hippocampal CA3 pyramidal neurons basal (F_6,28_=33.28, *P*<0.001) and apical (F_6,28_=17.93, *P*<0.01) dendrites show a significant decrease in their length in animals that were exposed to uCMS. Treatment with KET (*P*<0.001), IMP-KET (*P*<0.001) and FLX-KET (*P*<0.001) recovered basal dendritic length, whereas FLX-KET was able to induce a significant increase in basal dendritic length compared with FLX alone (*P*<0.01). Neither IMP nor FLX alone increased CA3 basal dendritic length significantly when compared with uCMS animals. Regarding the apical dendritic tree, treatment with KET (*P*<0.01), IMP-KET (*P*<0.01), FLX alone (*P*<0.01) and FLX-KET (*P*<0.01) induced recovery, whereas treatment with IMP alone failed to increase CA3 apical dendritic length significantly. Both IMP-KET and FLX-KET promoted a higher regrowth than IMP (*P*>0.001) and FLX (*P*>0.001; Figures 5m and p). Exposure to uCMS induced an increase in dendritic length in NAc medium spiny neurons (F_6,28_=24.73 *P*<0.001), and this feature was reversed within all treatment groups (all *P*<0.01, Figure 5a).

A significant decrease in spine density (SD) was found in basal (F_6,28_=22.11, *P*<0.001) and apical (F_6,28_=6.86, *P*<0.05) IL dendrites of uCMS animals. Treatments with KET (*P*<0.001), IMP (*P*<0.001), IMP-KET (*P*<0.001), FLX (*P*<0.001) and FLX-KET (*P*<0.001) increased SD in IL basal dendrites; FLX-KET significantly increased SD in basal dendrites when compared with FLX alone (F_1,28_=6.86, *P*<0.05). Treatment with FLX (*P*<0.001) and FLX-KET (*P*<0.001) increased SD in apical dendrites. No significant increase of SD in apical dendrites was found in animals treated with KET or IMP alone and in animals treated with IMP-KET. Regarding spine morphology, mushroom spines are decreased in uCMS animals both in apical (F_6,28_=5.712, *P*<0.001) and basal (F_6,28_=2.668, *P*<0.001) dendrites when compared with control animals. Treatment with KET (*P*<0.001), IMP-KET (*P*<0.001) and FLX-KET (*P*<0.001) reverted this pattern in basal and apical IL dendrites. No significant increase in mushroom spine population was found in animals treated with IMP or FLX alone; although the treatment with IMP-KET and FLX-KET caused a significant rise in mushroom spine population in apical and basal dendrites when compared with IMP (*P*<0.05) and FLX (*P*<0.01) alone (Figures 5e, f, h and i).

Hippocampal dentate gyrus neurons in uCMS untreated animals have significant lower dendritic SD (F_6,28_=7.613, *P*<0.05) when compared with control animals. Treatment with FLX (*P*<0.01) and FLX-KET (*P*<0.01) significantly increased SD. There was no significant increase in SD in animals treated with KET or IMP alone or with IMP-KET. When evaluating the spine morphology, specifically the more developed mushroom spines, uCMS animals present also less number of mushroom dendritic spines in dentate gyrus neurons (F_6,28_=8,162, *P*<0.05) (*t*=5.267, *P*<0.001) but treatment with KET (*t*=5.477, *P*<0.001), IMP (*t*=4.339, *P*<0.01), IMP-KET (*t*=5.903, *P*<0.001), FLX (*t*=4.205, *P*<0.01) and FLX-KET (*t*=5.359, *P*<0.001) restored mushroom spines numbers (Figures 5k and l).

In the CA3, neurons from uCMS animals displayed a significantly lower dendritic SD in apical (F_6,28_=73.57, *P*<0.001) and basal (F_1,28_=22.12, *P*<0.001) dendrites, which were recovered upon treatment with all the tested drugs (*P*<0.001 to all). Addition of KET to FLX treatment statistically increases spine density (F_1,28_=6.86, *P*<0.05) in apical dendritic tree. The population of mushroom dendritic spines is decreased in untreated uCMS animals both in apical (F_6,28_=23.86, *P*<0.001) and basal (F_6,28_=6.882, *P*<0.01) CA3 dendrites. In apical dendrites, treatment with KET (*P*<0.001), IMP (*P*<0.05), IMP-KET (*P*<0.001) and FLX-KET (*P*<0.001) caused an increase in mushroom spine population. Both IMP-KET (*P*<0.01) and FLX-KET (*P*<0.001) induce higher mushroom dendritic spine population. In basal dendrites, treatment with KET (*P*<0.01), IMP-KET (*P*<0.01) and FLX-KET (*P*<0.01) increased the amount of mushroom spines (Figures 5n, o, q and r).

Exposure to uCMS induced a significant increase in SD in medium spiny neurons of the NAc (F_6,28_=7.297, *P*<0.01) and this feature was reversed in the treatment with IMP, IMP-KET, FLX-KET (*P*<0.01) and FLX (*P*<0.05). Regarding spine morphology, uCMS promoted a decrease in the population of mushroom spines (F_6,28_=9.094, *P*<0.001) and all treatments significantly increase mushroom spine compared with the untreated uCMS animals (*P*<0.001). Importantly, the animals treated with antidepressant and ketamine (IMP-KET and FLX-KET) presented a significant increase of mushroom spine population when compared with those who were only given antidepressant (IMP or KET; Figures 5b and c).

## Discussion

The use of KET in subanesthetic dose with antidepressant effect is described both in animal models of stress and in MD patients.^13, 24, 25^ Classic antidepressants are used with success in our animal model, the uCMS.^26^ In our study, we aim to test the effect of KET on the response of classic antidepressants. The effect of KET alone is described in the literature, although there is a lack of understanding regarding the synergetic effect of initial antidepressant treatment with KET. Therefore, we propose to investigate the effect of KET on the response of classic antidepressants. In the literature, the methodology described for the administration of KET is very heterogeneous; it goes from single to multiple doses, the latest one given either daily or in intermittent days. When designing the protocol of antidepressant treatment we decided to use a multiple continuous method with a short duration (3 days).

The SCT was used as a behavioral hallmark for recovery from depressed phenotype. The first evaluation occurred at the seventh day of treatment and indicated that only animals treated with KET (either alone or in combination with other antidepressant) display behavioral rescue of anhedonia. After one additional week of treatment, at the 14th day, all treated groups show reversion of anhedonia, except the one only treated with KET, which showed a reversion of the positive behavioral effect. In our results, we can see the specific effect of the interaction of KET added to antidepressant treatment on the results of the treated group KET-FLX. The effect of KET alone in anhedonia does not last until the 14th day, but when given with FLX, it enhances it.

The second hallmark of this model is learned helplessness, a measure of behavior despair. There was an increase in latency to immobility time in all treated animals but the analysis of total immobility time shows that the effect of KET alone is not detected 2 weeks after being administrated. Again, when KET is given with FLX, it is evident that there is an increase in latency time compared with the antidepressant alone.

The analysis of anhedonia and despair in our model showed that combining KET with other treatments seems to anticipate the increase in sucrose preference and to improve the response. KET possibly is acting as an initial booster and keeps the antidepressant response. However, when given alone it failed to show a robust result.

We also assessed anxiety and show that the combination of KET with FLX or IMP was able to significantly reverse the anxiety trait. Again, there seems to be no anxiolytic effect after 2 weeks of KET given alone. Some literature reports long-lasting anxyolitic effect of KET given in a single dose to patients with depressive disorders^27^ and in animal models when given in multiple doses.^28^ However, we observed a positive effect when given with antidepressants. When given together with the antidepressants, beneficial effect of KET extends beyond the mood domain and also potentiates the anxiolytic effects of SSRIs and tricyclic antidepressant drugs.

The positive effect of initial dual therapy with KET and other antidepressant, concerning behavioral recovery in our animal model, is a faster initial increase in sucrose preference and a more robust response after 2 weeks of treatment not only in anhedonia but also in behavioral despair and anxiety.

MD disorder and stress disorders have been associated with impaired functional connectivity,^29, 30^ as a result of dysfunction occurring by several pathways. To evaluate the impact of KET in neurotransmission, we have analyzed the levels of both GLU and also we evaluated the dendritic architecture. We assessed the levels of GLU in regions relevant for depressive-like behavior. Our major finding was that NAc, a brain region implicated in anhedonia,^26, 31^ was the only region where KET, in association with other antidepressants, triggered an increase in the levels of GLU and ASP suggesting that this particular effect is ascribed to GLU metabolism. The failure to observe significant changes in GLU or ASP levels in other brain regions probably relates to temporal dynamics of the experimental design. However, literature reports that both acute stressors and chronic stressors increase basal release and increased presynaptic reuptake of GLU, but most measurements were performed using mycrodialysis or synaptossomal analysis, thus reflecting extracellular contents, not the overall content of GLU. There is evidence that KET acts as a noncompetitive and nonselective high-affinity NMDA antagonist also on GABAergic neurons and may rapidly increase GLU release, for example, in the PFC.^32, 33^ Although there is clinical evidence that treatment for depression increases the release of GLU in the NAc,^34^ other studies using KET in individuals with MD were not consistent in showing an association between therapy and changes in neurotransmitter content.^35, 36^ Present data suggest changes in glutamatergic transmission, particularly in the NAc, a mechanism through which KET helps restore mood.

Through the analysis of neuronal architecture, we confirmed the impact of uCMS on shrinkage of dendritic arborizations in the hippocampus and in the IL area of the PFC, and dendritic hypertrophy in NAc medium spiny neurons.^7, 8, 26, 37^ Globally, groups treated with antidepressant and KET showed a recovery of the morphology of their dendritic trees, with a more consistent response in FLX than IMP. Yet, the most striking changes were noticed in dendritic spines; we not only found that the addition of KET to the antidepressant treatment boosted a significant recovery in spine density when compared with antidepressants alone, but also with greater spine maturation. KET is known to have effects in synaptic plasticity: the fast-acting antidepressant action of KET is known to be dependent on rapid protein synthesis (namely of BDNF, PSD-95, GLuR1 and synapsin 1) and activation of intracellular pathways such as mTOR, all of them physiologically relevant for synaptic plasticity mechanisms, neuronal growth, differentiation and synaptogenesis.^38, 39, 40, 41^ Although the specific role of mTOR in NAc is not fully understood in depression, the effect of mTOR modulation is well studied in addiction. Therefore, we can speculate the existence of some similarity between addiction development and in antidepressant action. Indeed, the use of rapamycin not only blocks establishment of addiction, but also the antidepressant effect of KET.^42^ The acute and lasting effect of KET in neuronal remodeling might be one of the reasons why it improves behavioral markers of recovery in our experiment. The particular case of combined treatment in increasing more mature dendritic spine population shows that processes related to synaptic functioning and rewiring have an important role in behavioral recovery.

In summary, the present data show that KET has the potential to improve the action of classic antidepressants in brain regions affected by uCMS, a validated animal model of depression. The addition of KET to antidepressants accelerate recovery from anhedonia. The specific addition to FLX improves not only anhedonia but also behavioral despair. Despite lack of robustness in neurochemical data, we show improvement of glutamatergic profile in an area related to anhedonic behavior. The effects in neuronal morphology show a potential mechanism for improvement in synaptic connectivity in brain areas known to be affected and responsible for behavioral hallmarks in MD. These results show that KET has potential to improve the initial response of MD treatment.
